# Acral Subcorneal Hematoma Mimicking Melanoma: Dermoscopic Features and Exploratory Digital Color Analysis

**DOI:** 10.7759/cureus.109272

**Published:** 2026-05-20

**Authors:** Jesús Iván Martínez-Ortega, Sri Naidnur

**Affiliations:** 1 Histology, Autonomous University of Nuevo Leon, Monterrey, MEX; 2 Dermatology, Dermatology Institute of Jalisco, Zapopan, MEX; 3 Dermatology, Lake Erie College of Osteopathic Medicine, Greensburg, USA

**Keywords:** acral hematoma, acral melanoma, acral pigmented lesions, dermoscopy, scratch test, subcorneal hematoma

## Abstract

Acral pigmented lesions pose a diagnostic challenge due to the overlap between benign entities, such as subcorneal hematoma, and acral melanoma. Dermoscopy is a key noninvasive tool that aids in differentiation by revealing subsurface structures not visible to the naked eye.

We report the case of a 54-year-old man presenting with an asymptomatic acral pigmented macule on the volar aspect of the finger. Clinically, the lesion appeared dark reddish-brown, raising concern for a melanocytic process; however, dermoscopic evaluation revealed a homogeneous, predominantly red, structureless pattern without melanocytic features. Based on these findings, a subcorneal hematoma was diagnosed, and a conservative approach was adopted.

To further explore the discrepancy between clinical and dermoscopic appearance, exploratory digital image analysis was performed. RGB histogram analysis demonstrated a predominance of the red channel in both clinical and dermoscopic images, with a greater relative contribution under dermoscopy. Additional evaluation using the CIELAB color space confirmed the presence of red chromatic components and revealed a shift in relative chromatic contrast between the lesion and surrounding skin. These findings are exploratory and serve to support, rather than establish, the observed optical phenomenon.

This case suggests that dermoscopy enhances the visibility and relative contrast of pre-existing chromatic components through optical modulation, rather than generating new information. The observed clinical-dermoscopic discrepancy highlights the role of optical factors and contrast modulation in color perception and underscores the value of dermoscopy in increasing diagnostic confidence and potentially avoiding unnecessary biopsy in appropriate clinical contexts.

## Introduction

Acral pigmented lesions represent a well-recognized diagnostic challenge due to an overlap between benign and malignant entities, particularly melanocytic and non-melanocytic conditions such as subcorneal hematoma [1,2]. Accurate differentiation is critical, as these entities carry markedly different clinical implications, ranging from self-limited processes to potentially life-threatening malignancy.

Dermoscopy plays a central role in the evaluation of acral lesions by enabling the visualization of subsurface structures that are not readily apparent to the naked eye [3]. Certain dermoscopic patterns may aid in distinction; for example, the parallel ridge pattern has been associated with high specificity for acral melanoma [4], whereas non-melanocytic lesions, such as subcorneal hematoma, typically demonstrate homogeneous pigmentation without melanocytic structures [1,2]. However, variability in dermoscopic presentation has been described, and not all lesions conform to classic patterns, which may complicate clinical decision-making [5].

Subcorneal hematoma is a benign condition resulting from hemorrhage confined to the stratum corneum, most commonly associated with mechanical trauma, although a history of trauma is not always identified [1,5]. Dermoscopically, these lesions may exhibit a range of colors, including red, brown, and black hues, reflecting differences in the age and organization of hemorrhagic material [1,2,5]. However, the focus of the present case is not the spectrum of subcorneal hematoma itself, but rather the observed discrepancy between clinical and dermoscopic color appearance.

Dermoscopy enhances the visualization of subsurface chromatic components by reducing surface reflection and improving light penetration [3,6]. This optical effect may reveal underlying hematic structures that are not readily perceptible on clinical inspection, resulting in differences between naked-eye and dermoscopic appearance.

Herein, we present a case of acral subcorneal hematoma that appeared dark clinically but demonstrated a homogeneous red pattern on dermoscopy. We further explore this clinical-dermoscopic discrepancy using a digital image analysis to illustrate how optical factors and relative chromatic contrast influence color perception.

## Case presentation

A 54-year-old man with no significant past medical history presented for the evaluation of an incidentally noted pigmented lesion on the volar aspect of the left second finger at the level of the distal interphalangeal joint. The lesion was asymptomatic, with no associated pain, bleeding, or reported changes in size or color. No clear history of preceding trauma was elicited.

**Figure 1:**
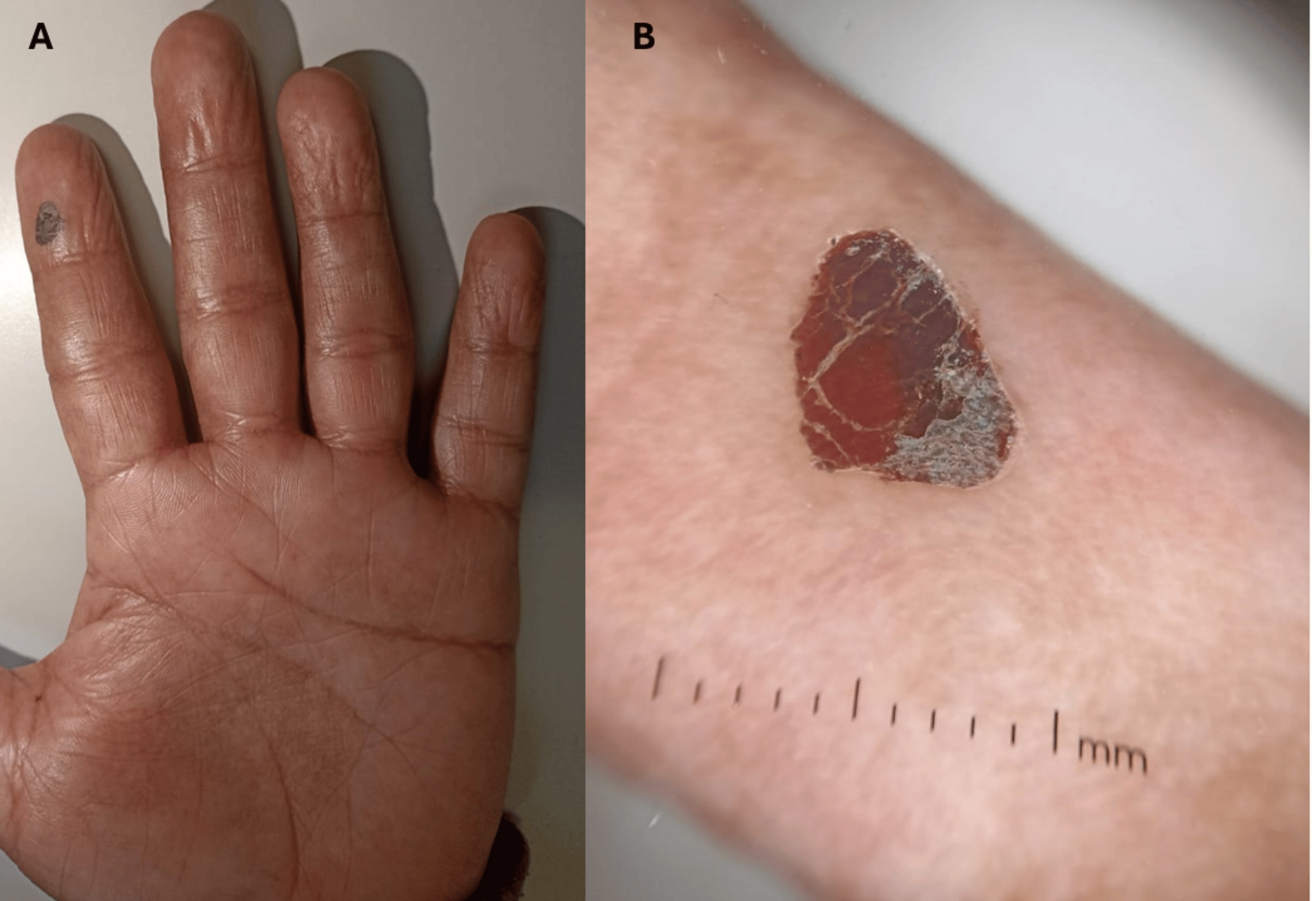
Clinical and dermoscopic appearance. (A) Clinical image of the acral lesion on the volar aspect of the finger, appearing as a dark reddish-brown macule. (B) Dermoscopic image demonstrating a homogeneous, structureless lesion with predominant red pigmentation and absence of melanocytic features.

On physical examination, a solitary acral macule measuring approximately 5 × 7 mm was observed. The lesion was polygonal in shape with relatively well-defined borders and appeared dark reddish-brown on clinical inspection (Figure 1A).

Dermoscopy using a polarized handheld dermatoscope (DermLite DL4; Aliso Viejo, CA, USA) revealed a predominantly red homogeneous pattern without melanocytic features, including the absence of a pigment network, parallel ridge pattern, or fibrillar pattern (Figure 1B).

**Figure 2:**
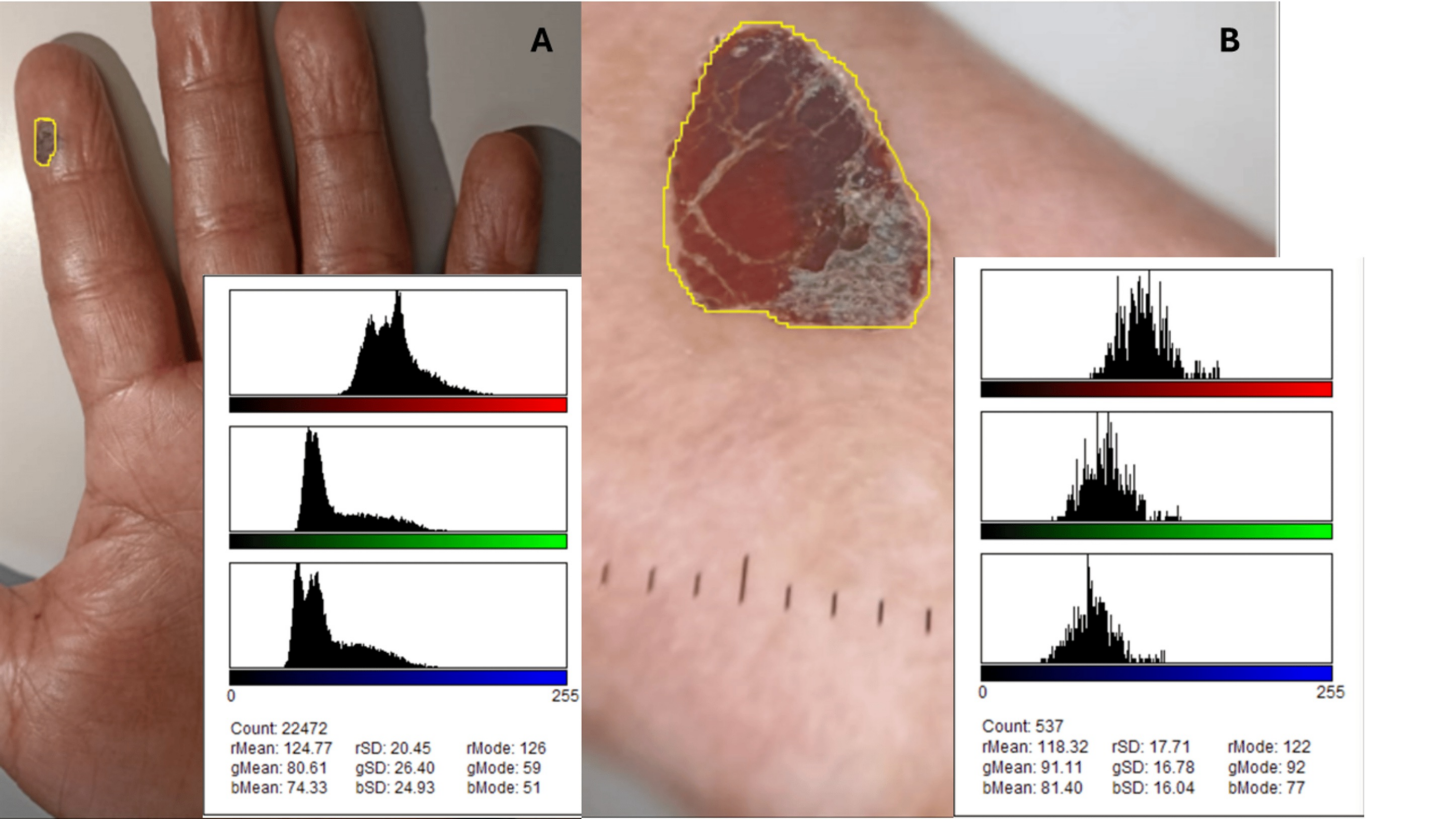
RGB histograms of the lesion in clinical (A) and dermoscopic (B) images. Regions of interest (ROI) within the lesion are outlined in yellow. Corresponding RGB histograms show the distribution of color components, with a relative predominance of the red channel in both settings and an increased contribution under dermoscopy. These findings support the presence of underlying hematic content despite the clinically darker appearance. Image processing and visualization were performed using Fiji (ImageJ; National Institutes of Health, USA).

To further evaluate the clinical-dermoscopic discrepancy, an exploratory digital image analysis was performed using region-of-interest (ROI) sampling of the lesion. RGB histogram analysis was used to assess the distribution of red, green, and blue channel intensities in the clinical (Figure 2A) and dermoscopic (Figure 2B) images.

**Figure 3:**
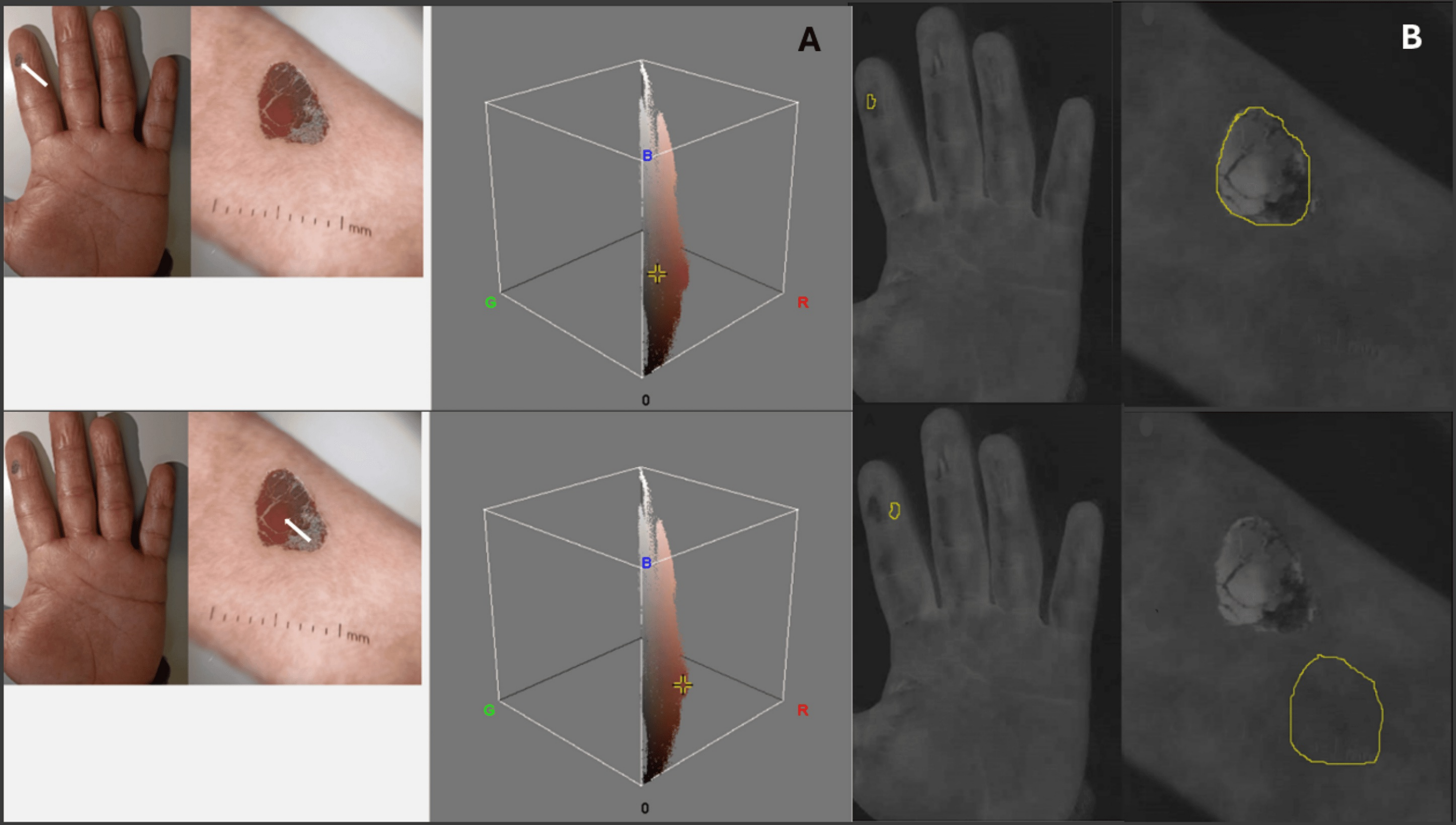
Point-based color inspection and CIELAB a* channel visualization. (A) Clinical and dermoscopic images showing the regions of interest (ROI) selected for point-based color sampling (white arrows). Corresponding three-dimensional color plots illustrate the distribution of color components within the selected ROI. An increased relative red contribution is observed in the dermoscopic image compared with the clinical image. (B) ROI-based visualization of the lesion and adjacent skin in the CIELAB a* channel, illustrating the spatial distribution of red chromatic components. Yellow outlines indicate the sampled regions of interest. Image processing and visualization were performed using Fiji (ImageJ; National Institutes of Health, USA).

Point-based color inspection was additionally performed using selected sampling points within the lesion to assess the local chromatic variation between clinical and dermoscopic images (Figure 3A), along with the region of interest (ROI)-based visualization in the CIELAB a* channel in both clinical and dermoscopic images to illustrate the spatial distribution of red chromatic components relative to the adjacent skin (Figure 3B).

The RGB histogram analysis demonstrated predominance of the red channel in both images despite the clinically darker appearance. Clinical and dermoscopic images were analyzed using standardized image captures available from routine clinical documentation. Exploratory ROI-based color analysis was performed in Fiji (ImageJ; National Institutes of Health, USA) without formal exposure or white-balance normalization. In the clinical image, mean values were R = 118, G = 91, and B = 81 (R/(R+G+B) = 0.41), while in the dermoscopic image, values were R = 124, G = 80, and B = 74 (R/(R+G+B) = 0.47), indicating an increased relative contribution of the red channel under dermoscopy. Further analysis using the CIELAB color space demonstrated positive a* values in both images, confirming the presence of underlying red chromatic components. The lesion showed an increase in a* values from the clinical image (a* = 11.3) to the dermoscopic image (a* = 22.5), consistent with enhanced visualization of hematic content.

A comparison with the adjacent normal skin demonstrated a shift in the relative chromatic contrast. In the clinical image, the perilesional skin showed higher a* values (a* = 25.7) than the lesion, whereas dermoscopy showed the lesion exhibiting higher a* values than the surrounding skin (a* = 14.9). These findings (Table 1) suggest that dermoscopy enhances relative chromatic contrast rather than simply increasing absolute color intensity.

**Table 1:** Digital color analysis of lesion and perilesional skin in clinical and dermoscopic images. Quantitative color analysis was performed using regions of interest (ROI) selected from the lesion and adjacent normal skin. RGB histogram values (red, green, and blue channels) and their relative contribution (R/(R+G+B)) were calculated to assess the channel dominance. An additional analysis in the CIELAB color space was performed to evaluate perceptual color differences, where the a* axis represents the green-red component, with positive values indicating increasing red contribution. A comparison between clinical and dermoscopic images demonstrates both the presence of underlying red chromatic components and a shift in the relative chromatic contrast between the lesion and surrounding skin.

Modality	Region	R	G	B	R/(R+G+B)	a* (CIELAB)
Clinical	Lesion	118	91	81	0.41	11.3
Clinical	Adjacent normal skin	-	-	-	-	25.7
Dermoscopy	Lesion	124	80	74	0.47	22.5
Dermoscopy	Adjacent normal skin	-	-	-	-	14.9

Based on the absence of melanocytic structures and the presence of a homogeneous hematic pattern, a diagnosis of subcorneal hematoma was favored. Given the benign dermoscopic appearance, a conservative approach with clinical follow-up was adopted. Although histopathologic confirmation was not obtained, complete spontaneous resolution of the lesion at one-month follow-up without residual pigmentation further supported the diagnosis of subcorneal hematoma.

## Discussion

Subcorneal hematoma shows a range of dermoscopic features that may overlap with melanocytic lesions, contributing to diagnostic uncertainty [1,2]. In previous series, the most common color pattern has been reported as red-black (approximately 40-45%), followed by brown, red, and black hues in varying proportions [1,2]. Homogeneous pigmentation represents the most frequently observed pattern, reported in approximately 53-65% of cases, followed by globular and ridge-associated patterns [1,2].

In the present case, a predominantly homogeneous, structureless pattern was observed, without evidence of melanocytic features. This finding is consistent with previous descriptions and supports the concept that the presence of a homogeneous pattern, regardless of the exact hue, represents a key dermoscopic clue favoring a nonmelanocytic process.

The diagnostic challenge arises from an overlap with acral melanoma, particularly given that hemorrhagic lesions may occasionally exhibit ridge-associated pigmentation. The parallel ridge pattern has been reported to demonstrate a specificity approaching 99% for acral melanoma [4], and its presence typically warrants biopsy. However, subcorneal hematoma may exhibit variable dermoscopic patterns, and clinical history is not always reliable, as many patients do not recall preceding trauma [1,5].

In this context, dermoscopy plays a critical role in guiding management decisions. In cases lacking melanocytic structures and demonstrating a homogeneous pattern, a conservative approach may be appropriate. When diagnostic uncertainty persists, simple bedside procedures, such as the paring (scratch) test, may help confirm a superficial hemorrhagic process [5]. In the present case, the absence of melanocytic criteria and lack of a parallel ridge pattern supported clinical observation without the need for invasive intervention.

Dermoscopy allows the visualization of subsurface morphologic structures not readily appreciable to the naked eye by reducing surface reflection and enhancing light penetration into the epidermis and superficial dermis [7,8]. At the stratum corneum level, differences in the refractive index between air and keratinized tissue result in the reflection and scattering of incident light, contributing to the clinically darker or less transparent appearance of the skin [7,8]. Dermoscopic techniques overcome this limitation using mechanisms, such as immersion interfaces and polarized light, which reduce surface glare and allow preferential detection of backscattered light from deeper layers [7,8].

This optical principle explains the clinical-dermoscopic discrepancy observed in this case, in which the lesion appeared dark clinically but showed a predominantly red appearance with increased visibility of red chromatic components under dermoscopic evaluation (Figures 1-3). These observations support the concept that dermoscopy may enhance the visibility and relative contrast of pre-existing subsurface chromatic components through optical effects, such as reduced surface reflection and altered light propagation [7-10].

In the present case, this phenomenon was further explored using digital image analysis. Initial RGB histogram analysis demonstrated a relative predominance of the red channel in both clinical and dermoscopic images, despite the clinically darker appearance. However, RGB values alone are highly dependent on illumination conditions and do not directly reflect perceptual color relationships [10].

To address this limitation, an additional analysis was performed in the CIELAB color space, which approximates human color perception. In this system, the a* axis represents the green-red component, with positive values indicating increasing red contribution. The lesion demonstrated positive a* values in both clinical and dermoscopic images, confirming the presence of underlying red chromatic components consistent with hemoglobin-containing structures (Figure 3, Table 1).

Interestingly, a comparison with the adjacent skin revealed a shift in the relative chromatic distribution. While perilesional skin showed higher a* values than the lesion on the clinical image, this relationship was reversed under dermoscopy, where the lesion showed higher a* values (Figure 3, Table 1). These findings suggest that dermoscopy does not simply increase the absolute color intensity, but rather enhances the relative chromatic contrast between the lesion and surrounding tissue.

This observation highlights that perceived color is influenced not only by the absolute channel intensity but also by contextual contrast. Accordingly, the use of both RGB histograms and perceptually oriented color models, such as CIELAB, provides complementary information, allowing a more nuanced interpretation of dermoscopic color changes.

Recent evidence suggests that morphologic characteristics may be more critical than color in dermoscopic diagnosis, as diagnostic accuracy can be maintained even in grayscale images [9]. However, this does not diminish the potential complementary role of chromatic information in specific clinical contexts. In the present case, the absence of melanocytic structures remained the primary diagnostic clue, while enhanced visualization of red chromatic components under dermoscopy likely served as an adjunctive feature supporting the recognition of a hematic process [9,10]. Thus, dermoscopic color enhancement may contribute not as an isolated diagnostic criterion, but as a contextual aid that increases confidence in pattern interpretation and lesion characterization.

**Figure 4:**
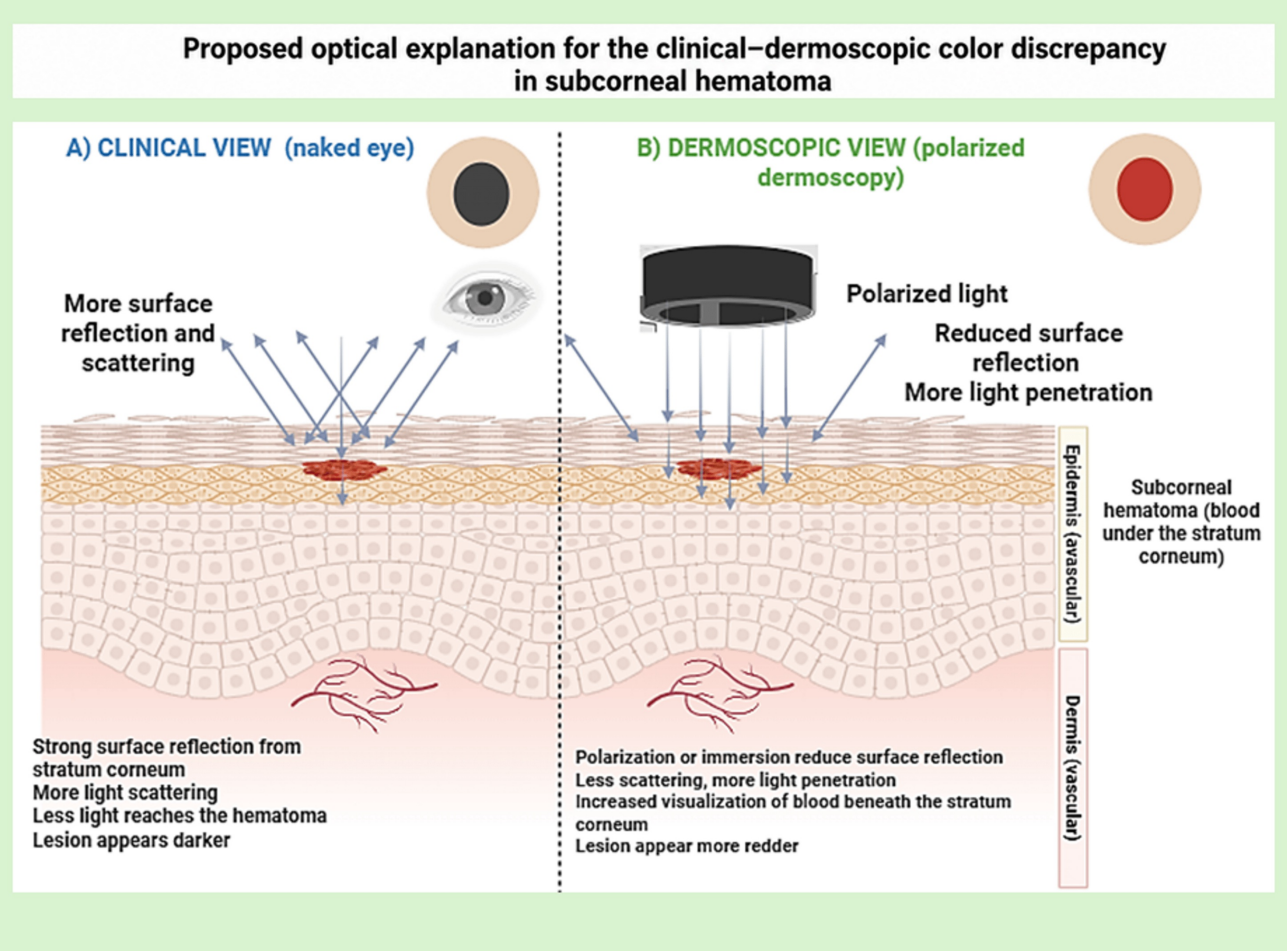
Proposed optical explanation for the clinical-dermoscopic color discrepancy in subcorneal hematoma. A schematic summarizing the proposed optical mechanisms underlying the different clinical and dermoscopic appearances of subcorneal hematoma. On naked-eye examination, increased surface reflection and light scattering at the stratum corneum may reduce visualization of superficial hematic material, contributing to a darker appearance. Under polarized dermoscopy, reduction of surface reflection and altered light propagation may enhance the visibility and relative contrast of subsurface blood components, resulting in a more prominent red appearance. This figure is intended for explanatory purposes only. Created with BioRender.com.

Together, these observations suggest that dermoscopy functions not merely as a magnification tool, but as an optical system that enhances both structural and chromatic perception through improved subsurface visualization and contrast modulation (Figure 4).

Several limitations should be acknowledged. Histopathologic confirmation was not obtained, as the lesion demonstrated benign clinical and dermoscopic features and subsequently resolved completely during follow-up, making invasive confirmation difficult to justify clinically. In addition, digital color analyses presented here are exploratory and descriptive and should not be interpreted as proof of a specific optical mechanism. Serial short-term follow-up with sequential image acquisition may have provided additional insight into temporal chromatic changes known to occur in subcorneal hematoma, including progression from red-purple to brown-black and yellow hues. Previous studies have shown that sequential observation, with or without paring tests, may help support the diagnosis of subcorneal hematoma in clinically equivocal cases [5].

## Conclusions

Subcorneal hematoma may present with variable dermoscopic patterns; however, a homogeneous, structureless appearance without melanocytic features remains a key diagnostic clue supporting a benign process. In acral locations, where melanoma exclusion is critical, dermoscopy plays a central role in guiding clinical decision-making and may reduce the need for unnecessary biopsies. This case suggests that dermoscopy may function not merely as a magnification tool, but also as an optical system that enhances visualization of subsurface structures by reducing surface reflection and modulating relative chromatic contrast. The discrepancy between clinical and dermoscopic color appearance underscores the importance of considering both structural patterns and optical principles in dermoscopic interpretation.

Exploratory digital image analysis further demonstrated the presence of red chromatic components that were less perceptible on clinical inspection but became more evident under dermoscopic evaluation. Together, these exploratory observations reinforce the value of dermoscopy as a noninvasive diagnostic tool and suggest that subtle variations in color perception may reflect underlying optical interactions relevant to lesion characterization.
